# Comparison of the Efficacy and Safety of Intravitreal Conbercept with Intravitreal Ranibizumab for Treatment of Diabetic Macular Edema: A Meta-Analysis

**DOI:** 10.1155/2020/5809081

**Published:** 2020-03-23

**Authors:** Xiaolei Sun, Jingjing Zhang, Jingyi Tian, Shijiu Chen, Fanxing Zeng, Gongqiang Yuan

**Affiliations:** ^1^Department of Medical College, Qingdao University, Qingdao, China; ^2^Shandong Eye Hospital, State Key Laboratory Cultivation Base, Shandong Provincial Key Laboratory of Ophthalmology, Shandong Eye Institute, Shandong First Medical University & Shandong Academy of Medical Sciences, Jinan, China

## Abstract

**Methods:**

Relevant studies were identified through systemic searches of PubMed, Embase, Cochrane Library, Ovid, CNKI, and Wanfang database up to 28 February 2019. Changes in central retinal thickness (CRT) in μm and best-corrected visual acuity (BCVA) in logMAR equivalents at 1, 3, and 6 months after initial treatment were performed by pooled analysis. Adverse events (AEs) were evaluated.

**Results:**

Eight articles involving 588 patients with DME were identified for this meta-analysis. The results showed that IVC significantly improved BCVA compared with IVR at 6 mo (SMD = −0.74 95% CI: −1.28 to −0.2; *p*=0.029) in patients with DME. IVC was superior to IVR in reducing central retinal thickness (CRT at 1 mo (*p* < 0.0001), 3 mo (*p*=0.025), and 6 mo (*p*=0.019)) from baseline with statistical significance. For AEs, the pooled results showed that no significant difference in the risk of intraocular pressure increased (OR = 1.71; 95% CI: 0.55 to 5.25; *p*=0.352) or conjunctival hemorrhage (OR = 0.89; 95% CI: 0.34 to 2.34; *p*=0.65) between two groups.

**Conclusions:**

This meta-analysis showed that IVC trended to be more effective than IVR in terms of functional and anatomic outcomes for treating DME.

## 1. Introduction

As a common manifestation of diabetic retinopathy (DR), diabetic macular edema (DME) is the foremost cause of central vision loss, and even blindness, which greatly influences patients' life quality [1]. For DR patients aged 20 to 79 years, the global prevalence for DME is 6.8% [2]. It has been reported that the prevalence of DME was related to the duration of the diabetes [1]. In patients with type 2 diabetes, the 10y incidence of macular edema was up to 14%, and 29% of type 1 progressed into DME over a 25y period [3]. Hence, it is urgent to find safe and effective treatment of DME.

Laser has been the gold standard treatment for DME since laser could result in a 50% reduction in severe vision loss [4]. Nevertheless, laser therapy has ocular side effects like subretinal fibrosis and laser scars [5]. Recently, it was reported that the chronically elevated level of serum glucose could damage the retinal-blood barrier (RBB) and upregulate the level of vascular endothelial growth factor (VEGF) [6], which result in the development and progression of DME [7]. Thus, therapeutic approach by inhibiting VEGF may be provided as an effective treatment of DME. Intravitreal anti-VEGF agents could significantly improve the vision and anatomic outcomes in DME patients, and their long-term efficacy and safety have been proven in numerous randomised trials [8–11].

Ranibizumab (RBZ, Lucentis, Genentech, Inc., San Francisco, CA, USA), the first anti-VEGF agent approved by the FDA, is a humanized monoclonal antibody fragment, which could bind all active forms of VEGF-A [12]. The most ultramodern anti-VEGF drug is conbercept, also named KH902 (Chengdu Kanghong Biotech Co., Ltd., Sichuan, China), which is a recombinant fusion protein containing the second immunoglobulin (Ig) domain of VEGF receptor 1 (VEGFR1) and the third and the fourth Ig domains of VEGFR2 and the Fc region of human IgG [13]. Similar to aflibercept in structure, conbercept could bind to all isoforms of VEGF-A, VEGF-B, and placental growth factor (PlGF). In addition, conbercept exhibits a higher affinity to VEGF due to the addition of the fourth Ig-like domain of VEGFR-2 in the Fab fragment [14]. Its affinity to VEGF is 50 times that of bevacizumab and 30 times that of ranibizumab [13, 15]. There were only small-sized clinical studies that compared the efficacy of intravitreal conbercept (IVC) with intravitreal ranibizumab (IVR) in DME treatment, and the data suggested that both conbercept and ranibizumab were effective in treating DME and could achieve similar efficacy [16]. It has been reported that the combined application of IVC injection and surgery can inhibit the generation and leakage of new blood vessels by inhibiting the signal transduction pathway of VEGF and its receptors, effectively reducing the intraoperative bleeding in patients with retinopathy and reducing the difficulty of surgery. It significantly shortens the operation time and reduces postoperative retinal edema and the risk of retinal detachment [17]. The application of IVC before vitrectomy can significantly reduce the operation time and the incidence of adverse events during the operation (*p* < 0.05) [18]. The occurrence of this result may be closely related to the atrophy of new blood vessels after the injection of drugs. The atrophy of new blood vessels can not only reduce intraoperative bleeding, ensure a relatively good surgical field of vision, but also reduce tissue adhesion to a certain extent [18].

Although anti–vascular endothelial growth factor (VEGF) agents are mostly chosen as a first-line treatment, there is an important role for steroids in the treatment algorithm for DME. The potential role of intravitreal steroids in disease modification here has a significant rationale. Corticosteroids reduce not only leukostasis and inflammatory cytokine production, but also VEGF expression [19]. Several studies have indicated that steroids were effective and safe for diabetic macular edema (DME) eyes' treatment [20–25].

To date, no systematic review has discussed the therapeutic effect and safety of IVC versus IVR in DME. Therefore, we performed this meta-analysis to quantify the effect and safety of conbercept and ranibizumab in the treatment of DME.

## 2. Methods

### 2.1. Search Strategy

A systematic search was performed in electronic databases, including Wanfang, CNKI, PubMed, Embase, Ovid, and Cochrane library. The search terms were as follows: “conbercept,” “Ranibizumab,” “diabetic macular edema,” and their synonyms or similar words (from their inception to February 2019) with the search formula of (Conbercept) AND (Ranibizumab) AND (“DME” OR “diabetic macular edema”). The reference lists of included articles and relevant reviews were searched manually to find other potentially eligible studies.

### 2.2. Inclusion and Exclusion Criteria

For inclusion, articles were selected on the basis of the following criteria: (1) the study population included patients with DME; (2) the IVC was included as an intervention; (3) randomized controlled trials (RCTs), retrospective or prospective cohort study, and observational study; and (4) reported best-corrected visual acuity (BCVA) or central retinal thickness (CRT).

Studies were excluded if (1) combined with other diseases; (2) they were performed in pediatric patients (≤18 years old) and pregnant women; and (3) no full texts, full texts without raw data, review articles, and duplicate publications.

### 2.3. Data Extraction

Data collection and analysis were performed according to a standard Cochrane protocol [26]. Two authors independently reviewed and extracted the data, such as study design, number of patients, patient characteristics (including age and gender), duration of follow-up, and treatment outcome in terms of BCVA and CRT. Inconsistency between authors was resolved by referral to a third reviewer.

Data of BCVA and CRT were showed in terms of mean ± standard deviation (SD), in condition that the value of mean or SD was not provided in the article, we used Get Data software to estimate the mean and the SD from the reported graph. The Cochrane Collaboration's tool was applied to evaluate the risk of bias of each included study.

### 2.4. Statistical Analysis

STATA version 12.0 (STATA Corp, College Station, TX, USA) was used for the meta-analysis. For the outcomes of dichotomous data (frequency of adverse events (AEs)), odds ratio (OR) with 95% confidence interval (CI) was calculated, whereas continuous data (BCVA and CRT), standardized mean difference (SMD), and 95% CI were calculated. Between-study heterogeneity was assessed by *I* square test. If *I*^2^ exceed 50% (*p* < 0.01), heterogeneity was considered statistically significant, and random-effects model was applied. When no heterogeneity was detected or the heterogeneity was relatively small, fixed-effects model was used for the meta-analysis. Sensitivity analysis was performed to evaluate the influence of a single study on the overall estimate.

## 3. Results

### 3.1. Literature Selection

**Figure 1:**
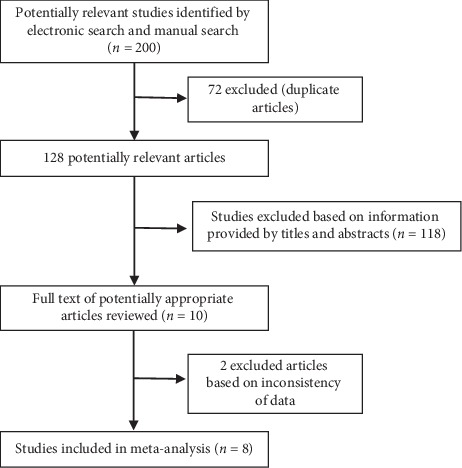
The process of selecting articles for the meta-analysis.

A total of 200 studies were initially identified according to the index words. After removal of duplicates (*n* = 72) and screening of abstracts (*n* = 118), 10 potential articles were assessed for eligibility. However, 2 studies were excluded because of inconsistency data. Ultimately, 8 articles [16, 27–33] published between 2016 and 2018 were included into the meta-analysis. The process of selecting articles for the meta-analysis is shown in Figure 1.

A total of 8 articles were amenable to meta-analysis, involving a total of 588 patients of whom 300 underwent IVC treatment and 288 underwent ranibizumab therapy. The sample sizes of different treatment groups varied from 50 to 110 subjects, and durations of follow-up varied from 3 to 12 month. The detailed characteristics of the included studies are described in Table 1.

### 3.2. Best-Corrected Visual Acuity (BCVA)

**Figure 2:**
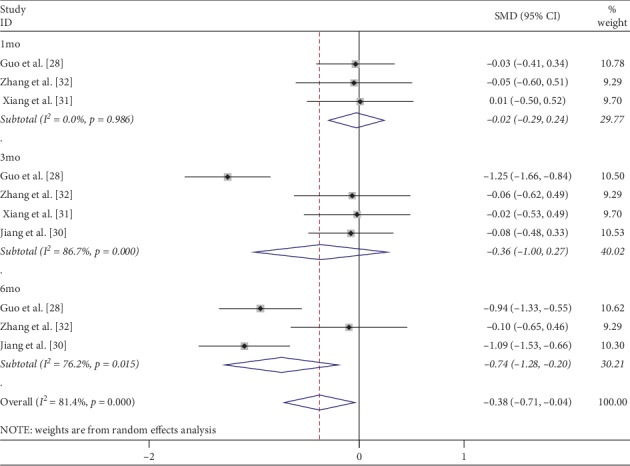
Forest plot of the mean change in BCVA at 1, 3, and 6 months after IVC treatment compared with that of IVR.

As the primary functional measure, BCVA was converted to logarithm of the minimum angle of resolution (logMAR) vision. The pooled analysis of the mean change in BCVA from baseline to 1, 3, and 6 mo after treatment were displayed in a forest plot (Figure 2). Three studies [28, 31, 32] reported data of BCVA at 1 mo after the initial treatment. The fixed-effects model analysis was conducted, because of the heterogeneity test results (*I*^2^ = 0%, *p*=0.986). No significant difference was found in BCVA between the IVC and IVR groups (SMD = −0.02; 95% CI: −0.29 to 0.24; *p*=0.855) (Figure 2). Four studies [28, 30–32] reported data of BCVA at 3 mo after the initial treatment and demonstrated significant heterogeneity among trials at any of these follow-up periods (*I*^2^ = 86.7%, *p*=0.0001). A random-effect model was used, and no significant difference was found in BCVA between the IVC and IVR groups at 3 mo (SMD = −0.36 95% CI: −1.00 to 0.27; *p*=0.261) (Figure 2). Three studies [28, 30, 32] reported data of BCVA at 6 mo after the initial treatment and demonstrated significant heterogeneity among trials at any of these follow-up periods (*I*^2^ = 76.2%, *p*=0.0015). The pooled results revealed that IVC significantly improved BCVA compared with IVR at 6 mo (SMD = −0.74 95% CI: −1.28 to −0.2; *p*=0.029) (Figure 2).

### 3.3. Central Macular Thickness (CMT)

**Figure 3:**
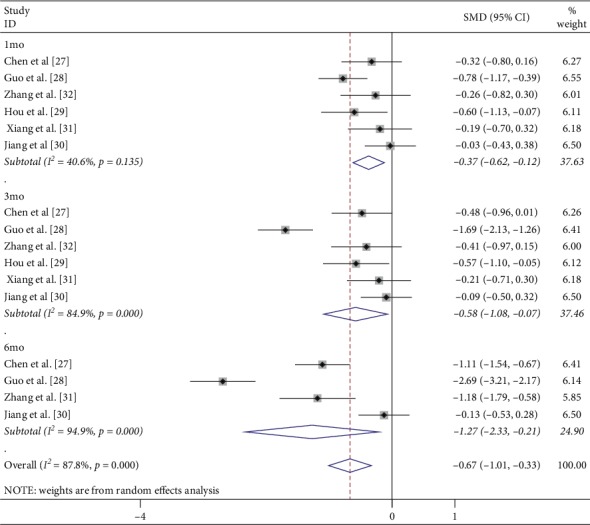
Forest plot of CMT at 1, 3, and 6 months after IVC treatment compared with that of IVR.

CMT represented the anatomic change after treatment. CRT was measured by Cirrus HD-OCT [16], 3D OCT-2000 (Japan, Topcon) [27], and other devices were not introduced in detail [28–33]. Six studies reported data on CMT at 1 mo after the initial treatment. Low heterogeneity was found among studies for this measure of effect (*I*^2^ = 40.6%, *p*=0.135), and a fixed-effect model was used. Both interventions resulted in decreased CMT, and IVC was significantly more effective at 1 mo compared with IVR (SMD = −0.38; 95% CI: −0.57 to −0.19; *p* < 0.0001) (Figure 3). Six and four studies reported data on CMT at 3 and 6 mo after the initial treatment, respectively. Among them, significant heterogeneity was observed (*I*^2^ = 84.9%, *p*=0.0001; *I*^2^ = 94.9%, *p*=0.0001, respectively). The random-effect model was used, and the pooled results revealed that IVC significantly reduced CMT compared with IVR at 3 mo (SMD: −0.58; 95% CI: −1.08 to −0.07; *p*=0.025) (Figure 3) and 6 mo (SMD: −1.27; 95% CI: −2.33 to −0.21; *p*=0.019) (Figure 3).

### 3.4. Adverse Events (AEs)

**Figure 4:**
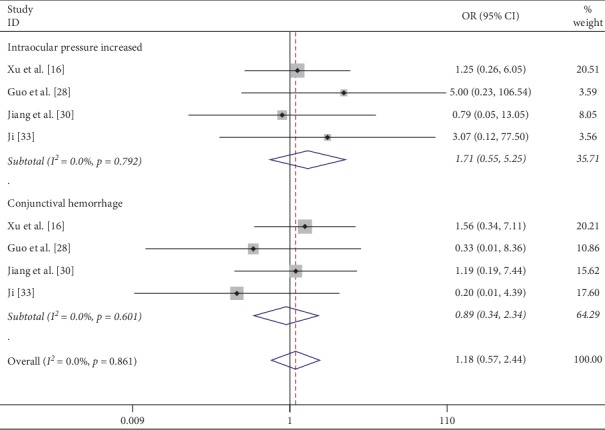
Forest plot of the incidence of increased intraocular pressure or conjunctival hemorrhage after IVC treatment compared with that of IVR.

Four studies demonstrated intraocular pressure increased after injection of conbercept and no heterogeneity among studies (*I*^2^ = 0%, *p*=0.792). Analysis using a fixed-effects model demonstrated no statistically significant difference between the IVC and IVR groups (OR = 1.71; 95% CI: 0.55 to 5.25; *p*=0.352) (Figure 4). Similarly, there was no significant difference in the increased risks of conjunctival hemorrhage (OR = 0.89; 95% CI = 0.34 to 2.34; *p*=0.65) (Figure 4), with no heterogeneity identified (*I*^2^ = 0%, *p*=0.861).

### 3.5. Sensitivity Analysis

**Figure 5:**
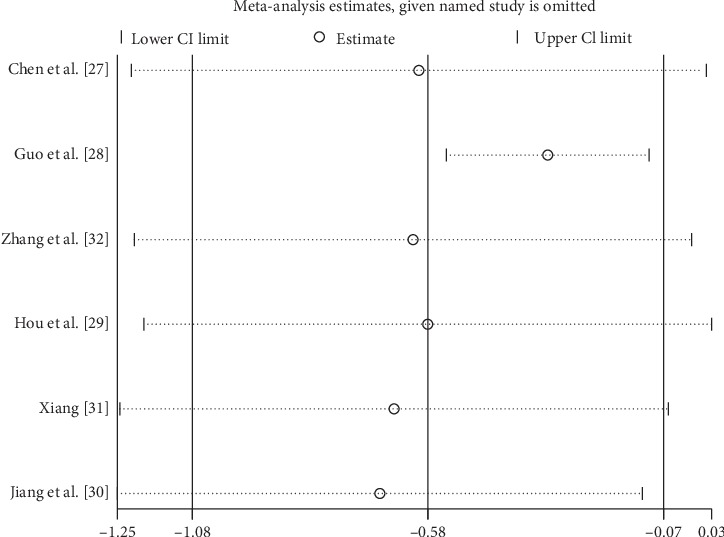
Sensitivity analysis for the CMT at 3 months after IVC treatment compared with that of IVR.

In the sensitivity analysis, we found that the removal of Guo et al. [28] decreased the heterogeneity with respect to BCVA or CMT at 3 mo and 6 mo after the initial treatment (Figures S1 and S2). The removal of any single study had a minimal impact on the value of BCVA or CMT, indicating the stability of the analysis (Figure 5).

## 4. Discussion

There is strong relationship between VEGF levels in both the anterior and vitreous chambers and DME severity [34, 35]. The addition of VEGF into a normal primate eye could cause the pathological changes of microaneurysm formation and vascular permeability increase, which are the hallmarks of diabetic retinopathy [36]. These researches have uncovered that VEGF may be a discernible target in the treatment of DME.

Ranibizumab, a recombinant humanized antibody fragment that binds vascular endothelial growth factor (VEGF), was the first drug approved for the treatment of DME [37]. Conbercept belong to the group of recombinant decoy receptors to VEGF. Conbercept have been shown to be effective and safe for DME treatment [38]. However, there are no systematic review discussed the therapeutic effect and safety of IVC versus IVR in DME.

In our study, we evaluated the efficacies and safety of IVC versus IVR in the treatment of DME based on eight studies including 588 patients. The results demonstrated that IVC could acquire significant improvement in BCVA at 6 mo (SMD = −0.74 95% CI: −1.28 to −0.2; *p*=0.029), as well as reduction in CMT at 1 mo (SMD = −0.38; 95% CI: −0.57 to −0.19; *p* < 0.0001), 3 mo (SMD: −0.49; 95% CI: −0.94 to −0.04; *p*=0.034), and 6 mo (SMD: −1.27; 95% CI: −2.33 to −0.21; *p*=0.019) compared with the treatment of IVR. Previous study included four retrospective studies and five RCTs with a total of 609 patients, suggesting that both IVC and IVR are effective in the therapy of diabetic macular edema and affirm that IVC presents superiority over IVR therapy in regard of CMT in patients with diabetic macular edema, but no statistically significant difference with regard to visual improvement [39].

BCVA, a primary measure of treatment efficacy, is an exceedingly important function outcome. In this meta-analysis, we found the improvements in BCVA did not vary significant between the IVC and IVR groups at 1 mo (*p*=0.855) and 3 mo (*p*=0.261) after the initial treatment. But, IVC could acquire significant improvement in BCVA at 6 mo (*p*=0.029). The results showed that with the increase of time, BCVA improvement in the conbercept group was better than that in the ranibizumab group. DME resulted in dysfunction of central and sharp vision. Our meta-analysis showed that there was no significant difference in the degree of visual acuity improvement between the two drugs during short-term treatment. As the disease progress, conbercept has a significant visual improvement in the treatment of 6 mo, which may be associated with more VEGF-binding sites than ranibizumab [40].

Intravitreal anti-VEGF drugs have good effect in reducing CMT and edema secondary to retinal vascular diseases, including short-term diabetic retinopathy [41, 42]. IVC could significantly reduce CRT at 1, 3, and 6 months, compared with IVR treatment. The results showed that conbercept could significantly reduce the retinal inflammation, decrease the content of VEGF in the tissues, and effectively reduce the macular edema.

Anti-VEGF drugs passed into the systemic circulation after delivering into the vitreous, which may cause cardiovascular events, infections and infestations, vascular disorders, and so on [43]. However, the number of observed adverse events was low in all studies. The incidence of increased intraocular pressure and conjunctival hemorrhage were similar in two groups.

Several limitations of the present meta-analysis could affect the final conclusion (1). Our meta-analysis was restricted to publications in English and Chinese language, without some potential eligible studies in other language included, which probably led to bias. (2) The results of this meta-analysis were based on studies with relatively small sample size and, therefore, should be interpreted cautiously. More well-designed and large-scale trials should be conducted to verify our results. (3) Some of the studies analysed have not be published in impact factor journals. More authoritative articles should be included in further study.

This meta-analysis is the first study to review the current evidences of published data regarding the use of conbercept versus ranibizumab in DME. Despite some limitations, the results of this meta-analysis are clinically useful and can offer some valuable, preliminary data on the clinical practice. However, the results of current literatures were uncompleted in all follow-up points, with the outcomes at 6 mo in most trials. Hence, more clinical studies in all follow-up phases should be required.

## Figures and Tables

**Table 1:** The detailed characteristics of the included studies.

	Included studies	Studies design	Treatment	Control	Sample size	Age, years	Sex (male/female)	Duration	Following
1	Chen et al. [27]	Observation study	Conbercept	Ranibizumab	34 : 34	NA	19/15 : 20/14	3 m	6 m
2	Xu and Rong [16]	Observation study	Conbercept	Ranibizumab	32 : 30	NA	NA	3 m	12 m
3	Guo et al. [28]	Observation study	Conbercept	Ranibizumab	55 : 55	61.35 ± 7.58 : 62.02 ± 6.48	28/27 : 29/26	6 m	6 m
4	Zhang et al. [32]	Observation study	Conbercept	Ranibizumab	25 : 25	51. 88 ± 10. 18 : 51. 60 ± 9. 70	13/12 : 11/14	3 m	6 m
5	Hou and Hu [29]	Observation study	Conbercept	Ranibizumab	29 : 29	55.9 ± 3.51 : 54.1 ± 3.87	19/10 : 17/12	3 m	3 m
6	Xiang [31]	RCT	Conbercept	Ranibizumab	30 : 30	58.97 ± 6.48 : 61.03 ± 7.12	14/16 : 13/17	3 m	3 m
7	Jiang et al. [30]	Observation study	Conbercept	Ranibizumab	53 : 42	60.75 ± 7.65 : 59.68 ± 7.02	24/29 : 18/24	3 m	6 m
8	Ji [33]	Observation study	Conbercept	Ranibizumab	42 : 43	57.0 ± 2.7 : 57.2 ± 2.6	21/21 : 23/20	NA	6 m

## Supplementary Materials

Supplementary Materials Figure S1: forest plot of the mean change in BCVA after removing Guo et al. [28]. Figure S2: forest plot of CMT after removing Guo et al. [28].
