# Supplementary figures and images for: Comparison of the Efficacy and Safety of Intravitreal Conbercept with Intravitreal Ranibizumab for Treatment of Diabetic Macular Edema: A Meta-Analysis
Source: J Ophthalmol. 2020 Mar 23;2020:5809081. doi: 10.1155/2020/5809081 (PMC7125465; doi:10.1155/2020/5809081)

Firgure S1

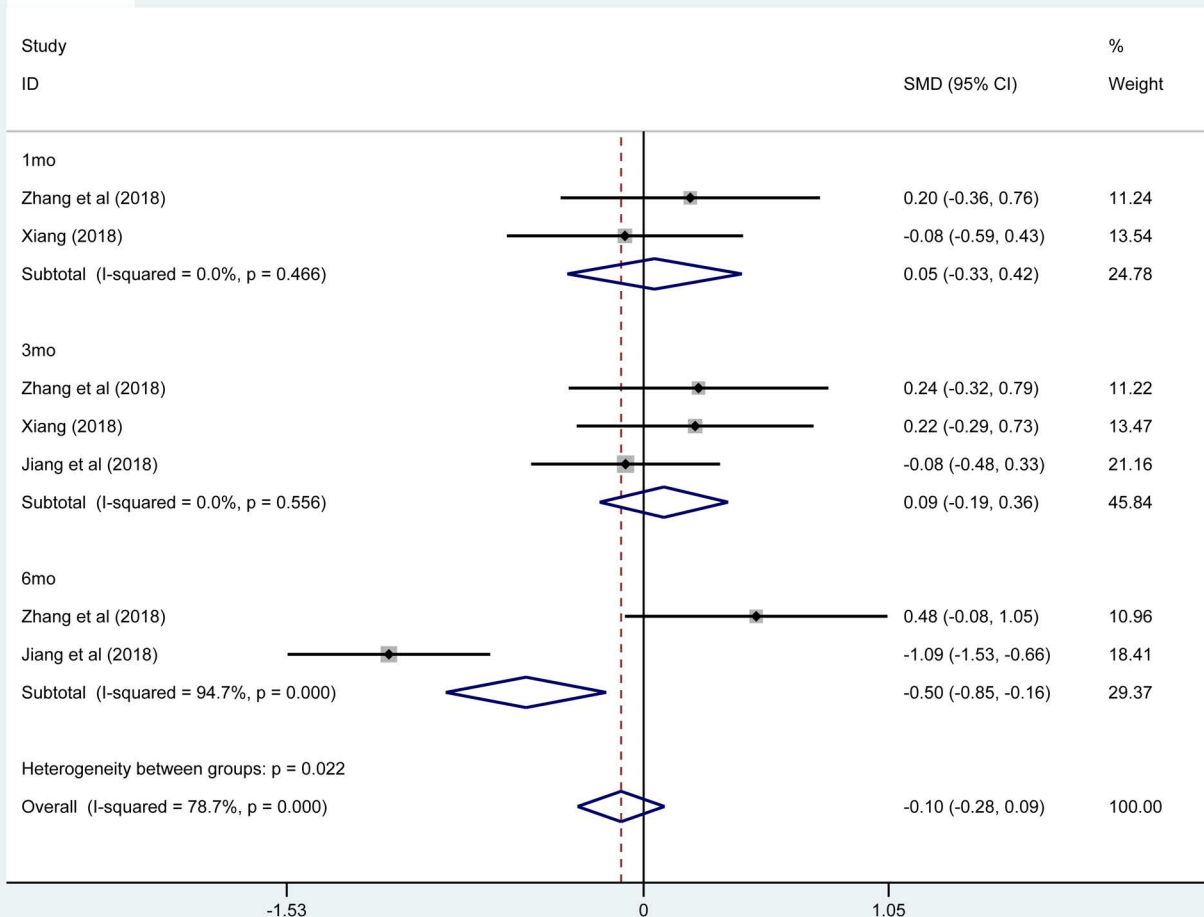

Figure S2

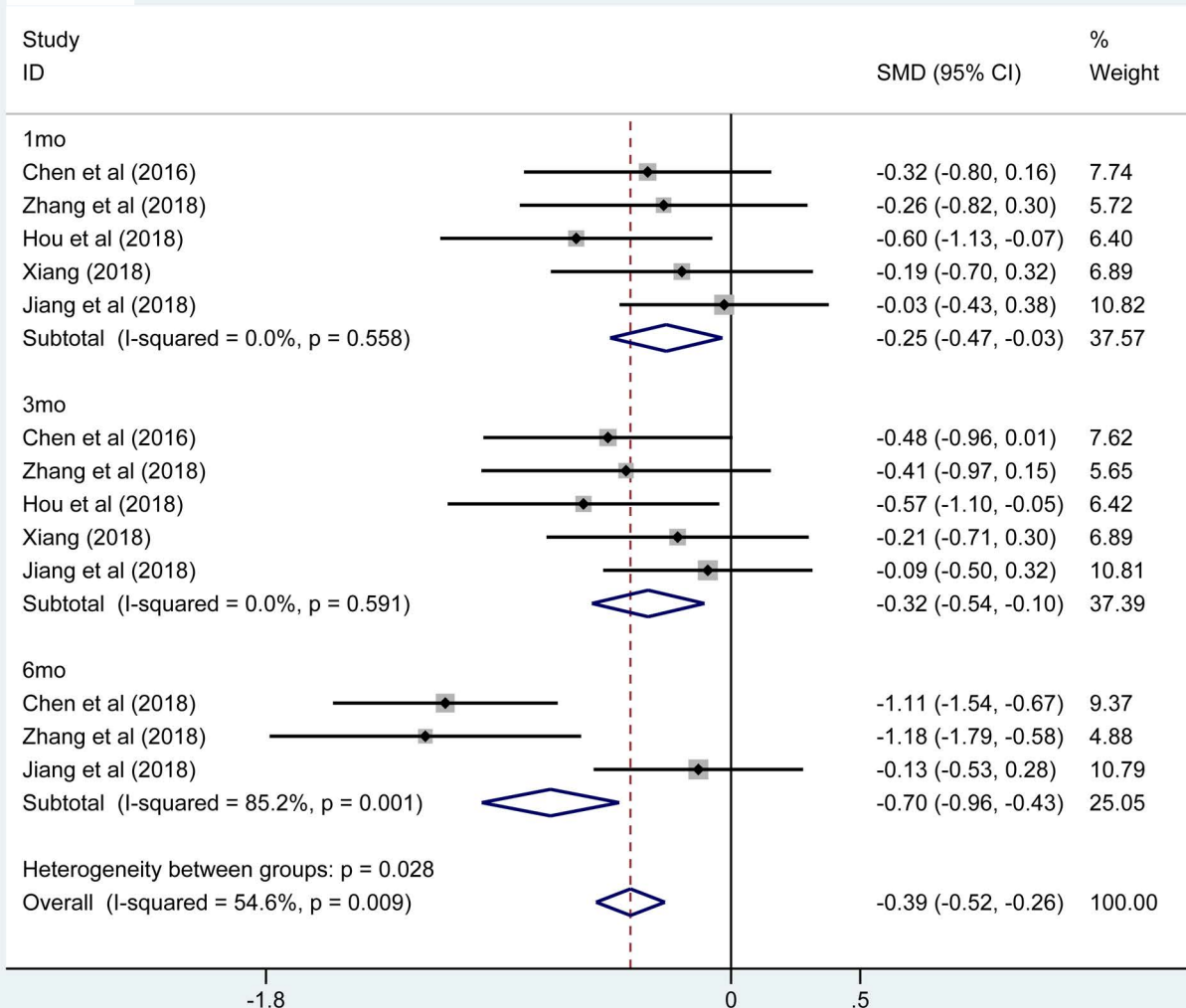

Supplement: Supplementary Materials — Figure S1: forest plot of the mean change in BCVA after removing Guo et al. [28]. Figure S2: forest plot of CMT after removing Guo et al. [28]. [file 5809081.f1.pdf]
